# Crystal, but not Clear

**DOI:** 10.31138/mjr.33.4.469

**Published:** 2022-11-23

**Authors:** Joydeep Samanta, Arghya Chattopadhyay, Ashish Jindal, Sanjay Jain

**Affiliations:** Clinical Immunology and Rheumatology Unit, Department of Internal Medicine, Post Graduate Institute of Medical Education and Research, Chandigarh, India

**Keywords:** hyperuricemia, crystal arthritis, diagnostics

**Figure 1.**
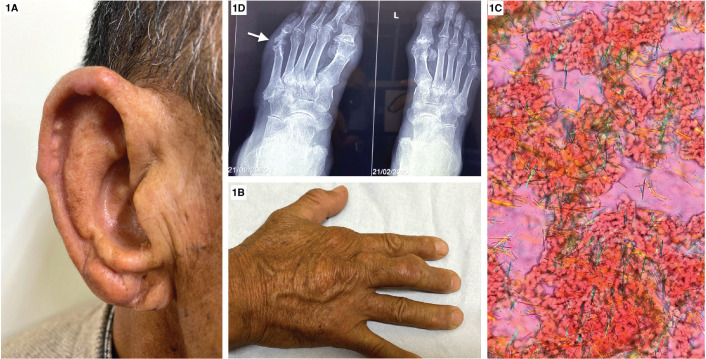
**(A)** Nodular swelling over ear helix. **(B)** Nodular swelling over finger. **(C)** Plain polarised microscopy showing negatively birefringent needle shaped uric acid crystals. **(D)** Radiograph of feet showing characteristic gouty erosions.

A 62-year-old man with a history of deforming polyarthritis for the last 10 years, presented with multiple nodular swelling over ears for six months. His history of joint symptoms was interspersed by multiple episodes of worsening which were being controlled with analgesics. Examination revealed multiple painless nodular swelling without erythema over ear helix and fingers (**Figure 1A,B**), hand and feet deformity, bilateral tender wrist joint without any tender or, swollen joint. Systemic examination was normal. Laboratory investigations revealed high inflammatory markers (ESR 30 mm/1^st^ hour, CRP 16 mg/Litre), negative rheumatoid factor and anti-CCP antibody and serum uric acid level of 7.2 mg/dl. Aspirate from the nodular swelling showed negatively birefringent needle-shaped uric acid crystals (**Figure 1C**) and radiograph of hands and feet showed characteristic sharp gouty erosions with overhanging edges (**Figure 1D**). A diagnosis of tophaceous gout with auricular tophi was made and started on allopurinol and colchicine.

Tophaceous gout is usually seen in chronic gouty arthritis, more commonly in the elderly. Incidence varies from 30% in the first 5 years to 72% in 20 years after the first acute attack in an untreated patient.^1^ Usual locations of tophi are fingers, toes, hands and feet; however, ear helix can rarely be involved (helical rims commonly, antihelix rarely). The usual presentation of auricular tophi is painless, well-circumscribed nodular lesion without any surrounding erythema, rarely with skin ulceration or, perforation of ear lobule. Hansen’s disease, amyloids, rheumatoid nodules, and elastotic nodules can have a similar presentation.^2^ Key points for differentiation are the presence of hypo aesthetic/anaesthetic skin lesion, neuropathy in Hansens’; painless and intensely pruritic papules, usually in concha in cutaneous amyloidosis; underlying diagnosis of rheumatoid arthritis with the usual location of nodules over extensor surface of the joints in rheumatoid nodules. Correct diagnosis is very important considering that it is responsive to urate lowering therapy.
